# Knowledge, attitudes, and practices related to family planning and gender equity among husbands of adolescent girls in Niger

**DOI:** 10.1080/17441692.2019.1692890

**Published:** 2019-12-03

**Authors:** Paul J. Fleming, Holly Shakya, Madeline Farron, Mohamad I. Brooks, Giovanna Lauro, Ruti G. Levtov, Sabrina C. Boyce, Sani Aliou, Jay G. Silverman

**Affiliations:** a Health Behavior & Health Education, University of Michigan School of Public Health, Ann Arbor, MI, USA; b Center on Gender Equity and Health, University of California, San Diego, CA, USA; c Pathfinder International, Watertown, MA, USA; d Promundo, Washington, DC, USA

**Keywords:** Contraception, maternal health, masculinity, francophone, Niger

## Abstract

Despite having the highest fertility rate in the world, research on Niger men and family planning (FP) is limited. We collected survey data collected in the Dosso region of Niger in 2016 from 1136 men who are the husbands of adolescent girls. We report descriptive statistics, bivariate and multivariable logistic regression on three dichotomous outcomes: (a) knowledge of modern contraceptives, (b) beliefs that only husbands should make FP decisions, and (c) current FP use. About 56% had ever heard of the pill, 6% had ever heard of an intrauterine device, and 45% had ever heard of an injectable. In our multivariable analyses, we found: a man knowing at least one modern method was significantly associated with his age, wife’s education level, gender ideology, and wife’s say in healthcare decisions; men’s belief that men alone should make FP decisions was associated with husband’s Quranic education, gender ideology, and attitudes towards violence against women; men’s reports of adolescent wives’ current family planning use was associated with men’s Quranic education, women’s involvement in her own healthcare decisions, and belief that men alone should decide about family planning. Finding suggests that interventions should target aim to reduce gender inequities to increase family planning utilisation.

## Introduction

Of all countries in the world, Niger – a land-locked country in Francophone West Africa – has the highest total fertility rate at 7.6 births per woman and the highest adolescent birth rate at 202 births per 1000 women ages 15–19 (Institut National de la Statistique, 2013). Most girls in Niger marry in adolescence – 76% marry by the age of 18 – and face pressure from husbands and their community to bear children immediately and frequently (Institut National de la Statistique, 2013). The United Nations ranks Niger as one of the countries with the greatest gender inequities, ranked 158th out of 159 countries on their Gender Inequality Index (United Nations Development Programme, 2016). In this context, husbands often make fertility/contraceptive decisions for the couple due to extreme gender inequities (Nouhou, 2016). Only 6% of married adolescent women ages 15–19 use a modern contraceptive method (Institut National de la Statistique, 2013).

Despite having the highest fertility rate and some of the greatest gender inequities, there is a dearth of research on Nigerien men’s perception of their role in family planning decisions and their attitudes/practices related to family planning. The data available on men in the 2012 Niger Demographic and Health Survey (DHS) – a traditional source for this type of data in most countries – is extremely limited with many common family planning-related questions not asked, such as men’s attitudes and practices related to family planning or gender equity (Institut National de la Statistique, 2013).

There are a few other studies in Niger that touch on elements of gender norms and family planning; however, the research base is very limited. One study examined data from the 2006 Niger DHS women’s survey using an analytic sample of women who were sexually active, breastfeeding, and using some form of modern or traditional contraception. This analysis showed that 52% of women reported using lactational amenorrhea as their primary method of contraception and that those women were more likely to report that they do not discuss family planning with their spouses (Sipsma, Bradley, & Chen, 2013). Another study in Niger collected survey data from adult women (*n* = 200) and found that subjective norms related to contraceptive use (e.g. ‘My friends approve of me using family planning methods’) were significantly associated with contraceptive use among women with no education (Mayaki & Kouabenan, 2015). However, this study did not collect data from men. A recent study published in French conducted survey research with women ages 15–49 and found that highly religious Nigerien women looked to their husbands or male religious leaders to inform their own attitudes about birth control (Nouhou, 2016). To our knowledge, these are the only three peer-reviewed studies that have examined the role of men/husbands in family planning utilisation in Niger and none report data collected from men.

Research from other areas of Francophone West Africa is more robust and shows the important role husbands play in family planning usage. In Burkina Faso, husband’s approval of contraceptives is a key determinant in women adopting modern family planning methods (Adebowale & Palamuleni, 2014). Other researchers, using data collected from postpartum women in Burkina Faso found that some husbands refused to refrain from unprotected sex and thus should be incorporated into postpartum FP interventions (Rossier & Hellen, 2014). A comparative study of Burkina Faso, Benin, and Mali further confirms that husbands must be considered when looking at fertility decisions (Pearson & Becker, 2014). This study uses DHS data to demonstrate that less than half of couples with any unmet need had concordance about that need, including cases of husband-only unmet need. Considering husband-only unmet need could be an entry point for family planning for some couples (Pearson & Becker, 2014). Finally, studies in Togo (Arnold et al., 2016) and Mali (Kaggwa, Diop, & Storey, 2008) show that husband approval and partner communication are key to a woman’s modern contraceptive use. Taken together, these studies suggest that information on husbands is critical to understanding family planning utilisation in the region.

This paper reports on newly collected (2016) survey data from 1136 men who are the husbands of adolescent girls in the Dosso region of Niger. Specifically, we used this data to (1) describe husbands’ socio-demographic characteristics and attitudes related to gender inequities and family planning decision-making, and (2) identify which socio-demographic and attitudinal factors are associated with (a) knowledge of modern contraceptives, (b) beliefs that only husbands should make FP decisions, and (c) men’s report of adolescent wives’ current FP use. We chose these three dimensions of Family Planning because they represent knowledge, attitudes about men’s roles, and the current utilisation of contraceptives. We hypothesise that men with more gender inequitable attitudes will be more likely to report lower knowledge of modern contraceptives, more likely to report believe that the man alone should make FP decisions, and less likely to report current FP utilisation. As the first survey with men on this topic, the analyses are exploratory and provide a foundation for future work.

## Methods

### Study setting

Niger is a land-locked country in Francophone West Africa. While comprehensive data on Niger is limited, the Demographic and Health Survey (DHS) provides a source of information on socio-demographics, health, fertility, and gender equity (Institut National de la Statistique, 2013). Niger is a predominantly Muslim (99%) country with some receiving a Koranic religious education in addition to or in place of traditional schooling. The two primary ethnic groups are Hausa (53% of the population) and Zarma/Songhai (21%). Gender inequity in Niger is reflected by differences in educational attainment: only 14% of women in Niger are considered literate, compared with 42% of men; and 64% of women have never been to school compared to 37% of men (Institut National de la Statistique, 2013). Niger also has some of the highest rates of early marriage in the world with the average age of first marriage for women being 15.7 years old (Institut National de la Statistique, 2013). Not surprisingly, adolescent motherhood is very common: 40% of women have their first child between the ages of 15–18 (Institut National de la Statistique, 2013). Polygyny is also common within Niger among men with enough resources to support more than wife; roughly one-third (36%) of married women have co-wives (Institut National de la Statistique, 2013). Prior research in Niger has shown that polygamous couples are less likely to discuss or use FP (Peterson, 1999). Twenty-seven percent of men and 60% of women agree that there are times a wife deserves to be beaten (Institut National de la Statistique, 2013). There are no official statistics on rates of intimate partner violence in Niger. Estimates of lifetime prevalence of physical or sexual intimate partner violence from neighbouring countries range widely, from 12% in Burkina Faso and 16% in Nigeria, to 35% in Mali and 51% in Cameroon (UN Women, 2017). This confluence of factors – low levels of education, early marriage, and high fertility, among others – makes it important to better understand the husbands of adolescent girls to fill a gap in our understanding of family planning utilisation in the country.

### Data Collection

Data were collected across 48 villages clustered within the Dosso, Doutchi, and Loga districts in the Dosso region of Niger as part of the baseline data collection for a cluster randomised control trial evaluating Reaching Married Adolescents, a contraception and gender intervention that targets married adolescent girls, their husbands, and communities led by Pathfinder International. Villages were randomly selected among all of those meeting the following inclusion criteria: (1) having at least 1000 permanent inhabitants; (2) primarily Hausa or Zarma-speaking (the two major indigenous languages of Niger); and (3) including no known recent intervention specifically around family planning or female empowerment with married adolescent wives or their husbands.

Twenty-five married female adolescents ages 13–19 years from each of the 48 villages and their husbands were randomly selected (using a random number generator) from a list of all eligible married female adolescents provided by each village chief. Eligibility criteria for the married female adolescents include: (1) ages 13–19 years old; (2) married; (3) fluent in Hausa or Zarma; (4) residing in the village where recruitment was taking place with no plans to move away in next 18 months or plan to travel for more than 6 months during that period; (5) not currently sterilised; and (6) provided informed consent to participate. The only eligibility criteria for the men included: (1) be married to one of the wives interviewed, and (2) provided informed consent to participate. There were 1314 husbands and 1315 adolescent wives contacted to participate in the study. Of these, 1156 and 1157, respectively, met inclusion criteria, consented to participate, and provided survey data, resulting in a baseline participation rate of 88% for both males and females. No significant differences in wife age, husband age, or time spent away from the village were observed across those who did and did not participate suggesting our sample does not differ socio-demographically from the broader population of married adolescents.

Separate surveys with the young women and their husbands were conducted by sex-matched trained research assistants from the Dosso region who could fluently read and speak French and fluently speak Hausa and/or Zarma. Research assistants visited the randomly selected households and conducted a Household Recruitment Screener to confirm eligibility. If the household was found not to include an eligible wife and husband dyad, a randomly selected replacement was recruited in their place. Up to three visits were made to each of the selected participants, after which no additional efforts were made.

Surveys were administered in a private location (out of earshot of another person, a place the participant indicated as private, typically in an outside area) in the village. Surveys were conducted in either Hausa or Zarma language, depending on the participant’s language preference. The survey took approximately 40–60 min to complete and was administered using pre-programmed tablets. This paper focuses on data collected from the husbands using an analytic sample with complete data on the variables of interest (*n* = 1136). Surveys with husbands were conducted by trained male research assistants from the Dosso region.

### Measures

All measures and survey instruments were pilot tested and reviewed and approved by a team of local Nigeriens with understanding of the local cultural context.

*Dependent Variables:* Our dependent variables were: family planning knowledge, belief that men alone should make FP decisions, and current family planning use. All questions were either yes/no or agree/disagree. For family planning knowledge, we asked men whether they are aware (yes/no) of contraceptive pills, intrauterine device (IDU), contraceptive injections, or contraceptive implants. We constructed a variable called Knowledge of Modern Methods where a man is coded as yes if he reports knowing at least one of those methods. For the belief that men alone should make FP decisions, we asked men whether they agreed or disagreed that ‘Only the man should decide whether to use a family planning method to delay pregnancy.’ We use a dichotomous measure where 1 = Agree and 0 = No or Don’t Know. For current family planning use, we asked men ‘Are you or [index wife] currently doing something or using any method to delay or avoid getting pregnant?’ (yes/no). This included both modern methods and traditional methods, but only 8 men reported using a traditional method.

*Gender Equity Variables:* We used four different variables to assess factors related to gender inequities.

For men’s gender ideology (i.e. attitudes about the roles of men and women), we used 22 items from the Gender Equitable Men (GEM) Scale. This scale has been successfully used previously in neighbouring Mali which shares many cultural characteristics (Slegh, Barker, Toliver, Bah, & Keita, 2013). Men were read each statement (see Table 2) and asked whether they Agreed, Disagreed, Don’t Know, or Declined to Answer. Based on the pattern of responses, we recorded responses as 1 = Agreed, 0 = Disagreed/Don’t Know/Declined. We conducted analyses to ensure that the items hung together and dropped items that did not fit. Our analyses use a sum score (possible range: 0–22; higher score indicating more gender-equitable beliefs) and the resulting measure has a Cronbach’s alpha 0.83.

We measured men’s attitudes about acceptability of violence against women by asking ‘In your opinion, is a husband justified in hitting or beating his wife in the following situation’ about five specific situations: (1) If she goes out without telling him? (2) If she uses a family planning method without his permission? (3) If she argues with him? (4) If she refuses to have sex with him? (5) If she burns his food? In our analyses, we use a continuous measure of how many items they endorsed.

We measure whether wives have a say in health decisions by asking husbands, ‘Who usually makes decisions about health care for your wife/wives and children?’ Response options include: respondent, wife, respondent and wife jointly, respondent’s mother, respondent’s father, respondent’s co-wife, someone else. In our analyses, we use a dichotomous measure where 1 = wife or respondent and wife jointly make health decisions, and 0 = respondent or others make health decisions.

For the belief that men alone should make FP decisions, we asked men whether they agreed or disagreed that ‘Only the man should decide whether to use a family planning method to delay pregnancy.’ We use a dichotomous measure where 1 = Agree and 0 = No or Don’t Know. (Note: this is also one of our dependent variables).

*Socio-demographic Characteristics:* We collected socio-demographic data as part of the Household Survey from the head of household, most often the husband, but others including the female participant were often present and, in some cases, provided reports. We included husbands’ and wives’ ages in years, and husbands’ and wives’ education as a continuous measure from 0 to 3, with 0 representing no formal schooling, 1-incomplete primary, 2-completed primary, and 3-past primary. We also included a binary measure of Quranic education for both husbands and wives. We measured whether the wife reported working in agriculture. Family wealth was assessed using the standard household assets list which we summed for each item that was reported in the home: a watch, a mobile phone, a bicycle, a motorbike or scooter, a car or truck, or an animal-drawn cart. Finally, we included number of children born to that couple, number of other wives the husband had, language and district where the couple lived. Need for Family Planning was assessed by determining which men did not have a pregnant wife, and reported wanting another child but longer than 1 year from now.

### Analyses

We report descriptive statistics, bivariate, and multivariable analyses. Using SAS 9.4, we conducted logistic regression with the three dichotomous outcomes: (a) knowledge of modern contraceptives, (b) beliefs that only husband should make FP decisions, and (c) current FP use. The analyses for current FP use were limited to only the sub-sample of men with need for FP (*n* = 592). First, we conducted bivariate analyses between each outcome and each socio-demographic variable or gender equity variable. Then, we developed multivariable logistic regression models for each outcome with all socio-demographic variables and only those gender equity variables that had a significance level of *p* < 0.10 in the bivariate analyses. We used *p* < 0.05 as a cutoff for significance and report descriptive statistics (Tables 1 and 2), bivariate results (Table 3), and multivariable results (Table 4).

**Table 1.** Socio-demographic characteristics of husbands of adolescent girls in the Dosso Region of Niger, *N* = 1136.

Demographics	Mean	SD	% Yes/Agree
Husband’s age	25.53	5.31	
Index wife’s age	17.29	1.54	
Wife’s age at marriage	14.18	1.84	
Husband’s education level (continuous 0–3)	0.88	1.00	
Husband quranic education			33.0
Wife’s education level (continuous 0–3)	0.57	0.87	
Wife quranic education			25.2
Wife works in agriculture			39.1
Family wealth	2.06	1.16	
Number of children between index couple	0.94	0.96	
Number of wives	1.15	0.40	
Language of interview			
Hausa			32.2
Zarma			67.8
District			
Dosso			32.4
Doutchi			34.2
Loga			33.4
Gender equity and FP knowledge/attitudes			
GEM sum score	4.2	3.5	
# of items where VAW is appropriate	1.3	1.6	
Wife has a say in health care decisions			4.5
Believes only man should make FP decisions			68.1
*Contraceptive knowledge/attitudes/behaviours*			
Overall knowledge of at least one modern method			65.4
Ever heard of the pill			56.5
Ever heard of an IUD			6.4
Ever heard of injectables			44.9
Ever heard of implants			10.5
Ever heard of lactational amenorrhea (non-modern method)			11.2
Desired family size			
0–4 children			2.4
5–9 children			44.6
10–14 children			23.0
>14 children			7.7
Unsure			22.4
Need for family planning			52.1
Current contraceptive use with wife			7.6

**Table 2.** GEM scale items and violence against women attitudes.

GEM scale items	*n*	% Agree
Men should not bathe, feed or otherwise take care of children.	691	63.5
A woman should obey her husband in all things.	1035	95.0
A woman should tolerate violence to keep her family together.	992	91.0
A woman’s most important role is to take care of the home and cook for the family.	1039	95.3
A man should have the final word about decisions in the home.	1035	95.0
More rights for women mean that men lose out.	1001	91.8
There are times when a woman deserves to be beaten.	644	59.2
I think it is shameful when men engage in caring for children or other domestic work.	644	59.1
If another man in my community insults me, I will defend my reputation, with force if I have to.	905	83.1
Giving baths to children, changing children’s clothes, and feeding children are the mother’s responsibility.	1002	91.9
A woman should never question her husband’s decisions even if she disagrees with them.	902	82.8
Women are too emotional to be leaders.	945	86.8
It is natural and right that men have more power than women in the family.	1030	94.8
If a man cooks or cleans it is shameful for his wife.	928	85.3
My only role for caring for my children is as their financial provider.	870	80.1
Only when a woman has a child is she a real woman.	831	76.5
A real man produces a male child.	611	56.2
It is the man who decides if and when to have sex.	766	70.7
Men are always ready to have sex.	825	76.3
Men need sex more than women do.	822	76.0
A woman should not initiate sex.	695	64.2
A woman who has sex before she marries does not deserve respect.	950	87.6
*Violence against women attitudes* *In your opinion, is a husband justified in hitting or beating his wife in the following situations:*		
If she goes out without telling him?	307	28.3
If she uses a family planning method without his permission?	325	29.9
If she argues with him?	261	24.0
If she refuses to have sex with him?	211	19.5
If she burns his food?	280	25.8
Acceptable in at least 1 case	548	50.8

**Table 3.** Results from bivariate logistic regressions with family planning-related dependent variables: (1) FP knowledge (*N* = 1136), (2) Beliefs about FP decision-making (*N* = 1136), and (3) Current FP use (*N* = 592).

	(1) Knows at least 1 modern contraceptive		(2) Believes man alone should make FP decision		(3) Currently uses FP method with wife	
	UOR	95% CI	UOR	95% CI	UOR	95% CI
*Socio-demographics*						
Husband’s age	1.06	(1.03–1.08), *p* < 0.01	0.99	(0.96–1.01), *p* = 0.25	1.05	(1.00–1.10), *p* = 0.04
Wife’s age	1.09	(1.01–1.18), *p* = 0.03	0.89	(0.82–0.97), *p* < 0.01	1.15	(0.94–1.40), *p* = 0.17
Wife’s age at marriage	0.95	(0.88–1.01), *p* = 0.11	0.99	(0.92–1.06), *p* = 0.76	0.85	(0.72–0.99), *p* = 0.04
Husband’s education level (continuous 0–3)	0.90	(0.78–1.02), *p* = 0.11	0.83	(0.72–0.94), *p* < 0.01	1.21	(0.90–1.64), *p* = 0.21
Husband quranic education	1.60	(1.22–2.10), *p* < 0.01	1.79	(1.35–2.38), *p* < 0.01	1.64	(0.93–2.90), *p* = 0.09
Wife’s education level (continuous 0–3)	1.20	(1.01–1.41), *p* = 0.04	1.00	(0.85–1.18), *p* = 0.98	1.23	(0.88–1.73), *p* = 0.23
Wife quranic education	1.59	(1.18–2.15), *p* < 0.01	1.26	(0.94–1.71), *p* = 0.13	0.82	(0.42–1.60), *p* = 0.55
Wife works in agriculture	0.62	(0.48–0.80), *p* < 0.01	0.94	(0.73–1.23), *p* = 0.67	1.38	(0.76–2.49), *p* = 0.29
Family wealth	1.14	(1.02–1.27), *p* = 0.02	0.99	(0.89–1.10), *p* = 0.85	0.76	(0.59–0.99), *p* = 0.04
Number of children between index couple	1.26	(1.10–1.44), *p* < 0.01	0.88	(0.77–1.00), *p* = 0.05	1.50	(1.16–1.96), *p* < 0.01
Number of other wives	0.98	(0.72–1.35), *p* = 0.92	1.02	(0.74–1.41), *p* = 0.91	1.03	(0.50–2.13), *p* = 0.93
Language of participant is Zarma (ref = Hausa)	1.96	(1.47–2.60), *p* < 0.01	1.36	(1.03–1.81), *p* = 0.03	1.46	(0.82–2.58), *p* = 0.20
District of participant is Dosso (ref = Loga)	2.22	(1.64–3.02), *p* = 0.03	0.91	(0.67–1.23), *p* = 0.15	0.96	(0.45–2.09), *p* = 0.40
District of participant is Doutchi (ref = Loga)	2.71	(1.99–3.70), *p* < 0.01	1.21	(0.89–1.66), *p* = 0.08	1.60	(0.79–3.23), *p* = 0.09
*Gender Equity Variables*						
GEM Scale (higher = more equitable)	0.94	(0.91–0.98), *p* < 0.01	0.87	(0.83–0.90), *p* < 0.01	0.94	(0.86–1.04), *p* = 0.23
# of items where VAW is appropriate	1.23	(1.12–1.34), *p* < 0.01	1.33	(1.21–1.46), *p* < 0.01	0.99	(0.83–1.19), *p* = 0.91
Wife has a say in her health care decisions	0.26	(0.15–0.48), *p* < 0.01	1.00	(0.54–1.83), *p* = 0.99	3.16	(1.12–8.95), *p* = 0.03
Believes only man should make FP decisions	1.43	(1.10–1.87), *p* < 0.01	–	–	0.41	(0.23–0.73), *p* < 0.01

UOR = Unadjusted odds ratio. CI = Confidence interval.

*p < 0.01, +*p* < 0.05.

**Table 4.** Results from three multivariable logistic regressions with family planning-related dependent variables: (1) FP knowledge (*N* = 1136), (2) Beliefs about FP decision-making (*N* = 1136), and (3) Current FP use (*N* = 592).

	(1) Knows at least 1 modern contraceptive		(2) Believes man alone should make FP decision		(3) Currently uses FP method with wife	
	AOR	95% CI	AOR	95% CI	AOR	95% CI
*Socio-demographics*						
Husband’s age	1.05	(1.01–1.09), *p* = 0.01	1.01	(0.97–1.04), *p* = 0.72	1.03	(0.96–1.11), *p* = 0.44
Wife’s age	1.01	(0.89–1.15), *p* = 0.84	0.91	(0.80–1.03), *p* = 0.13	1.00	(0.75–1.34), *p* = 0.99
Wife’s age at marriage	0.96	(0.87–1.07), *p* = 0.44	1.00	(0.90–1.10), *p* = 0.92	0.97	(0.78–1.20), *p* = 0.75
Husband’s education level (continuous 0–3)	0.97	(0.81–1.15), *p* = 0.72	0.89	(0.75–1.05), *p* = 0.16	1.27	(0.87–1.87), *p* = 0.22
Husband quranic education	1.15	(0.80–1.64), *p* = 0.46	1.75	(1.21–2.51), *p* < 0.01	2.54	(1.17–5.53), *p* = 0.02
Wife’s education level (continuous 0–3)	1.35	(1.10–1.67), *p* < 0.01	1.10	(0.91–1.34), *p* = 0.34	1.08	(0.72–1.63), *p* = 0.71
Wife quranic education	1.05	(0.70–1.57), *p* = 0.80	0.89	(0.60–1.32), *p* = 0.55	0.51	(0.21–1.24), *p* = 0.14
Wife works in agriculture	0.75	(0.54–1.03), *p* = 0.08	1.01	(0.73–1.39), *p* = 0.95	0.89	(0.43–1.84), *p* = 0.74
Family wealth	1.09	(0.96–1.23), *p* = 0.20	0.91	(0.80–1.03), *p* = 0.13	0.81	(0.60–1.09), *p* = 0.16
Number of children between index couple	1.21	(0.97–1.49), *p* = 0.09	0.90	(0.74–1.10), *p* = 0.30	1.34	(0.87–2.06), *p* = 0.18
Number of other wives	0.83	(0.53–1.31), *p* = 0.42	1.09	(0.71–1.67), *p* = 0.69	0.55	(0.18–1.69), *p* = 0.30
Language of participant is Zarma (ref = Hausa)	1.52	(0.60–3.88), *p* = 0.38	1.66	(0.67–4.12), *p* = 0.27	0.46	(0.08–2.69), *p* = 0.38
District of participant is Dosso (ref = Loga)	1.92	(1.35–2.73), *p* = 0.10	0.75	(0.52–1.07), *p* = 0.79	1.02	(0.43–2.46), *p* = 0.26
District of participant is Doutchi (ref = Loga)	1.50	(0.58–3.88), *p* = 0.87	0.48	(0.19–1.22), *p* = 0.21	3.66	(0.56–23.77), *p* = 0.16
*Gender equity variables*						
GEM scale (higher = more equitable)	0.95	(0.91–1.00), *p* = 0.04	0.89	(0.86–0.93), *p* < .01	–	–
# of items where VAW is appropriate	1.11	(1.00–1.23), *p* = 0.05	1.25	(1.12–1.39), *p* < .01	–	–
Wife has a say in her health care decisions	0.42	(0.21–0.84), *p* = 0.01	–	–	4.20	(1.22–14.52), *p* = 0.02
Believes only man should make FP decisions	1.34	(0.98–1.82), *p* = 0.07	–	–	0.37	(0.20–0.70), *p* < 0.01

AOR = Adjusted odds ratio controlling for all other variables listed. CI = Confidence interval.

## Results

Socio-demographics and family planning knowledge/attitudes of husbands are presented in Table 1. Husbands of adolescent girls in the Dosso region of Niger had a mean age of 26 (SD: 5.31) and on average married their wife when she was fourteen years old. Most husbands had never attended school. On average, the husbands had had 1 child (SD: 0.96) with the index wife and several men had more than one wife at the time of data collection.

We found low knowledge of contraceptive methods among the husbands. About two-thirds (65%) of men had heard of at least one modern contraceptive method (i.e. pill, IUD, injection, or implant). About 56% had ever heard of the pill, 6% had ever heard of an intrauterine device (IUD), 45% had ever heard of an injectable, and 11% had ever heard of implants.

For attitudes related to gender inequities and family planning, we found that husbands often held views that emphasised their power in their relationship. For example, 50.8% of husbands endorsed at least one of five criteria where it would be acceptable to beat their wife and the average number of criteria endorsed was 1.3 (SD = 1.6). Only 5% of men said their wife has a say (i.e. jointly with her husband or independently) in health decisions for herself and her children. Over two-thirds of men (68.1%) felt that only men should decide about family planning decisions.

For Knowledge of Modern Contraceptives, the bivariate analyses demonstrate that men who know at least one method are significantly more likely to have more gender inequitable attitudes (Odds Ratio [OR]: 0.94, 95% Confidence Interval [CI]: 0.91–0.98), list a higher number of items where VAW is appropriate (OR: 1.23, 95% CI: 1.12–1.34), have a wife who does not have a say in healthcare decisions (OR: 0.26, 95% CI: 0.15–0.48), and believe that only the man should make FP decisions (OR: 1.43, 95% CI: 1.10–1.87). Our multivariable model showed that when controlling for all other items in the model, Knowledge of Modern Contraceptives was significantly associated with four variables: husband’s age, wife’s education level, GEM Sum Score, and Wife’s say in healthcare decisions. Every one-point increase on a husband’s GEM Sum Score, suggesting more gender-equitable attitudes, was associated with a 5% reduction in odds of knowing at least one modern method (95% CI: 0.91–1.00). Additionally, men who reported that their wife has a say in healthcare decisions had lower odds of knowing at least one method (OR: 0.42, 95% CI: 0.21–0.84). Results indicated that men who listed a higher number of items where VAW is appropriate and believed that only the man should make FP decisions may be more likely to know at least one modern method, but these results were marginally non-significant (*p* = 0.05 and *p* = 0.07, respectively). Husband’s age and wife’s education level remained significant in the multivariable model but all other significant variables in bivariate models became non-significant in the multivariable model.

We next focused on the outcome Beliefs that Only Man Should Make FP Decisions. In the bivariate analyses, this outcome was significantly associated with GEM Sum Score (OR: 0.87, 95% CI: 0.83–0.90), and Attitudes about VAW (OR: 1.33, 95% CI: 1.21–1.46). In the multivariable model controlling for all other variables, these two variables remained significant at *p* < 0.01. The only other significant variable was husband’s receipt of Quranic education. For every point increase in men’s GEM Score – suggesting more equitable attitudes – there was a corresponding 11% reduction in odds of believing that only men should make FP decisions (AOR: 0.89, 95% CI: 0.86–0.93). For each additional situation that husbands thought VAW was acceptable there was a 25% increase in odds that he believed only men should make FP decisions (AOR: 1.25, 95% CI: 1.12–1.39).

Finally, we examined factors associated with whether or not men reported using modern contraceptives with his wife. The bivariate models showed that the only gender equity variable associated with current contraceptive use was the belief that men alone should make FP decisions. In our multivariable model, this variable remained significant at *p* < 0.05. Controlling for other factors, husbands who believed that only men should be making FP decisions had 39% lower odds of reporting current contraceptive use (AOR: 0.61, 95% CI: 0.37–1.00, *p* = 0.049). Other variables significant in the multivariable model included husband’s Quranic education, number children between the couple, language of participant, and district where he lived. Finally, in multivariable regression, men’s actual current family planning use was associated with wife having a say in her health care decisions, and belief that men alone should decide about family planning. If the husband reported that his wife had a say in her healthcare decisions, then the husband was significantly more likely to report currently using a FP method (AOR: 4.20, 95% CI: 1.22–14.52). If a man believed he should make FP decisions, he was significantly less likely to report that he and his wife currently use a FP method (AOR: 0.44, 95% CI: 0.25–0.77).

## Discussion

These findings present new and novel data with husbands of adolescent wives in Niger – an understudied population – on their knowledge, attitudes and practices related to gender equity and family planning. We demonstrate that most of these husbands are supportive of gender inequitable attitudes and that knowledge of modern contraceptives, beliefs that husbands should make family planning decisions and current use of a family planning method are all associated with the included gender equity variables. In this section, we discuss the implications of these findings.

Our descriptive findings provide new information about husbands of adolescent girls in Niger. About half of men had heard of the pill or injectable (i.e. DepoProvera) – the two most common forms of modern contraceptives used in Niger (Institut National de la Statistique, 2013) – but more than one-third of husbands had never heard of any of these types of modern contraceptive methods. Given that 68% of husbands believe they should be making family planning decisions alone, there is much room for education specifically with men on contraceptive methods.

Our results also demonstrate the gender inequitable attitudes of husbands interviewed. The average GEM Score was 4.2 where a score of 22 would be the most equitable (83% scored 7 or below and would be categorised as ‘low equitable attitudes’). As a point of comparison, a recent survey using the GEM scale in Mali (including urban and rural men) showed that 37% of men in Mali had ‘low equitable attitudes’ (Slegh et al., 2013). Most husbands (51%) in our study felt there was at least one reason a woman should be beaten and only 4.5% of men said that their wife has a say healthcare decisions. Men with more inequitable GEM scores and who supported violence against partners were both more likely to say that they should be making family planning decisions. In total, over two-thirds of men felt that only men should make FP decisions. This finding is related to the strict separation of gender roles in Niger and is similar to other studies of men in the region (Fleming et al., 2015; Shattuck et al., 2011).

There were also some surprising findings related to gender equity and family planning. For example, for the factors associated with Knowledge of a Modern Method, men who held more equitable gender attitudes (higher GEM score) and who found VAW unacceptable were *less* likely to know about a modern method. This runs counter to previous studies that show that more equitable gender equity attitudes (i.e. higher GEM Score) are associated with higher levels of education (Barker et al., 2011; Fleming et al., 2015). These somewhat surprising relationships may be explained by the fact that husbands who believe that men should make FP decisions were *more* likely to have gender inequitable attitudes. This may suggest that men with gender-equitable attitudes are more willing to leave issues of FP to their wives and thus are not educated about modern contraceptives because they are not involved in that decision. While this explanation is plausible, it would need further research to confirm.

Two gender equity variables (belief that men should make FP decisions alone and wives having a say in their healthcare decisions) were significantly associated with current family planning use. These findings highlight that inequitable decision-making related to family planning or health more generally is linked to contraceptive use. It may be that men overall are less inclined to support FP use and so if they believe they should be making the decisions they make the decision to not use FP. Future qualitative research should explore these relationships further.

Previous research with the GEM Scale would suggest that more equitable attitudes among husbands are associated with more favourable reproductive health outcomes for their wives/partners, including contraceptive use (Fleming et al., 2018). The lack of significant results for this variable and the attitudes towards VAW may be due to the fact that most men held inequitable views so there were few men with equitable view to compare them to. Most of this prior research using the GEM scale has been conducted in settings with less extreme forms of gender inequities. Future research is needed to better understand how gender-equitable attitudes, as measured by the GEM scale, play a role in contraceptive use in a setting with extreme gender inequities.

These findings contribute to the body of literature demonstrating that family planning decisions are subject to gender and power dynamics that shape how decisions are made within a relationship (Blanc, 2001; Stephenson, Bartel, & Rubardt, 2012). Previous research has shown that men’s gender ideology (e.g. attitudes about appropriate roles/responsibilities for men and women) is influential on their willingness to use contraceptives with their partner such that men with more traditional attitudes supporting distinct roles and norms for men and women are less likely to support contraceptive use (Barker et al., 2011; Mishra et al., 2014; Stephenson et al., 2012). Additionally, men’s gender ideology influences the characteristics of their relationships. Men who support a more traditional masculine ideology with different roles for men and women are more likely to be less communicative (Barker et al., 2011), more violent (Harrison, O’Sullivan, Hoffman, Dolezal, & Morrell, 2006), and more concerned with being the primary or sole earner in the household (Connell, 1995). This evidence points to the importance of men’s gender ideology for their relationship dynamics and contraceptive decision-making behaviours.

Our findings suggest that interventions should address gender inequities by targeting both husbands and wives (Dworkin, Fleming, & Colvin, 2015). Our findings, along with findings from other studies (Fleming et al., 2018; Shattuck et al., 2011), suggest that gender-transformative interventions focusing on gender-power dynamics have potential to improve family planning utilisation (Barua, Pande, MacQuarrie, & Walia, 2004; Greene & Barker, 2011; Pachauri, 2014; Raj & McDougal, 2015; Raju, 2000; Silverman & Raj, 2014). Such interventions can work with both men and women (i.e. gender synchronisation) to question inequitable gender norms and change men’s beliefs to facilitate shared decision-making around family planning issues; they have been shown to help improve men’s support for contraceptive use (Fleming et al., 2018; Greene & Levack, 2010).

*Limitations.* While we present several novel findings, our results should be considered in light of several limitations. First, our data is cross-sectional and thus we cannot attribute causality to these relationships. Second, the GEM Scale was originally developed in Brazil and though it had a strong Cronbach’s alpha with our sample and has been used globally, it may not be the ideal way to measure gender ideology among this population. Third, our analyses do not include all possible variables that might be associated with family planning utilisation, therefore these results should be considered exploratory. Fourth, men’s decisions and perspectives on family planning are likely to be influenced by other social actors and future research should better examine how their opinions and behaviours are shaped by community elders, mothers-in-laws, or religious leaders. Finally, we relied on self-report for all of our findings. Given that family planning is a sensitive topic in this region, our results may reflect social desirability bias rather than men’s individual attitudes.

## Conclusions

Niger has the highest fertility rate in the world and women, men, and children’s wellbeing are being negatively affected. To improve overall health, it is important that women (and men) who have unmet need for contraceptives are able to limit or space their births. In Niger, husbands play a powerful role in limiting their wife’s ability to access modern contraceptives. Interventions with men are urgently needed to increase their knowledge of family planning methods and support their wife’s family planning decisions.

## Data Availability

The data that support the findings of this study are available on request from the corresponding author, PJF. The data are not publicly available due to the fact that the data contain information that could compromise the privacy of research participants.
